# Real-World Switching to Riociguat: Management and Practicalities in Patients with PAH and CTEPH

**DOI:** 10.1007/s00408-018-0100-3

**Published:** 2018-02-22

**Authors:** Henning Gall, Jean-Luc Vachiéry, Nobuhiro Tanabe, Michael Halank, Mauricio Orozco-Levi, Lisa Mielniczuk, MiKyung Chang, Kai Vogtländer, Ekkehard Grünig

**Affiliations:** 1 Department of Internal Medicine, Justus-Liebig-University Giessen, Universities of Giessen and Marburg Lung Center (UGMLC), Member of the German Center for Lung Research (DZL), Klinikstrasse 32, 35392 Giessen, Germany; 2 Département de Cardiologie, Cliniques Universitaires de Bruxelles, Hôpital Erasme, Brussels, Belgium; 3 Department of Respirology and Department of Advanced Medicine in Pulmonary Hypertension, Graduate School of Medicine, Chiba University, Chiba, Japan; 4 Medical Clinic 1/Pneumology, University Hospital Carl Gustav Carus, Dresden, Germany; 5 Hospital Internacional de Colombia, Fundación Cardiovascular de Colombia, Santander, Colombia; 6 University of Ottawa Heart Institute, Ottawa, ON Canada; 7 Bayer AG, Berlin, Germany; 8 Bayer AG, Wuppertal, Germany; 9 Center for Pulmonary Hypertension, Thorax Clinic at the University Hospital, Heidelberg, Germany

**Keywords:** Chronic thromboembolic pulmonary hypertension, Endothelin receptor antagonists, Phosphodiesterase type 5 inhibitors, Pulmonary arterial hypertension, Real-world evidence, Riociguat

## Abstract

**Purpose:**

A proportion of patients with pulmonary arterial hypertension (PAH) and chronic thromboembolic pulmonary hypertension (CTEPH) do not achieve treatment goals or experience side effects on their current therapy. In such cases, switching patients to a new drug while discontinuing the first may be a viable and appropriate treatment option. CAPTURE was designed to investigate how physicians manage the switching of patients to riociguat in real-world clinical practice. Observations from the study were used to assess whether recommendations in the riociguat prescribing information are reflected in clinical practice.

**Methods:**

CAPTURE was an international, multicenter, uncontrolled, retrospective chart review that collected data from patients with PAH or inoperable or persistent/recurrent CTEPH who switched to riociguat from another pulmonary hypertension (PH)-targeted medical therapy. The primary objective of the study was to understand the procedure undertaken in real-world clinical practice for patients switching to riociguat.

**Results:**

Of 127 patients screened, 125 were enrolled in CAPTURE. The majority of patients switched from a phosphodiesterase type 5 inhibitor (PDE5i) to riociguat and the most common reason for switching was lack of efficacy. Physicians were already using the recommended treatment-free period when switching patients to riociguat from sildenafil, but a slightly longer period than recommended for tadalafil. In line with the contraindication, the majority of patients did not receive riociguat and PDE5i therapy concomitantly. Physicians also followed the recommended dose-adjustment procedure for riociguat.

**Conclusion:**

Switching to riociguat from another PH-targeted therapy may be feasible in real-world clinical practice in the context of the current recommendations.

**Electronic supplementary material:**

The online version of this article (10.1007/s00408-018-0100-3) contains supplementary material, which is available to authorized users.

## Introduction

Pulmonary hypertension (PH) is a debilitating condition that is classified based on the underlying etiology into five categories, including pulmonary arterial hypertension (PAH; group 1) and chronic thromboembolic pulmonary hypertension (CTEPH; group 4) [1–3].

There are several approved treatments for PAH: endothelin receptor antagonists (ERAs), prostacyclin analogs or prostaglandin I2 (IP) receptor agonists, phosphodiesterase five inhibitors (PDE5i), and the soluble guanylate cyclase (sGC) stimulator riociguat [1, 3, 4]. For patients with CTEPH, the gold standard treatment is the potentially curative pulmonary endarterectomy (PEA) [1, 5, 6]. Medical therapy with riociguat remains the option for patients who are ineligible for surgery, or develop persistent/recurrent CTEPH [1, 6–15]. Balloon pulmonary angioplasty is an emerging, minimally invasive treatment currently under investigation [16].

For patients with PAH who do not achieve treatment goals, current European Society of Cardiology/European Respiratory Society (ESC/ERS) guidelines recommend double- or triple-sequential combination therapy [1]. In studies investigating escalation of treatment, the initial drug is routinely continued. However, in cases where patients do not respond to the initial therapy, there are no data on whether the additional clinical effect is based on the drug combination. As such, in some cases it may be better to discontinue the original agent before starting a new therapy [17].

This strategy of switching patients from one PH-targeted therapy to another is largely unexplored in clinical practice. Small studies and case reports have demonstrated positive outcomes after switching, but these have largely involved switching within a drug class, and were mainly due to lack of efficacy [18–20] or safety and tolerability [21, 22] of the former drug.

Riociguat is currently the only medical therapy approved for the treatment of both PAH and inoperable or persistent/recurrent CTEPH [23, 24]. Switching from a PDE5i to riociguat in PAH patients with an insufficient response to treatment has been explored in the RESPITE study. Results from this uncontrolled pilot study indicated that this may be a feasible and effective treatment strategy [25]. Subgroup analysis from a CTEPH early access study and a study of 23 patients switching from PDE5i to riociguat suggest that switching from off-label PH-targeted therapy to riociguat is well tolerated in patients with CTEPH [20, 26].

Despite promising preliminary data, little is known about how switching to riociguat is managed in clinical practice. The CAPTURE study was designed to investigate how and why patients with PAH and inoperable or persistent/recurrent CTEPH are switched from other PH-targeted therapies to riociguat in real-world clinical practice. Data from the study were also used to assess whether recommendations from the riociguat prescribing information were in line with real-world practice [23].

## Methods

### Study Design

CAPTURE (clinicaltrial.gov: NCT02545465) was an international, multicenter, uncontrolled, retrospective chart review that collected data from patients with PAH or inoperable or persistent/recurrent CTEPH, who switched to riociguat from another PH-targeted therapy.

### Patients

Male and female patients with PAH or inoperable or persistent/recurrent CTEPH who were switched to riociguat from another PH-targeted medical therapy and completed a 5-month documentation period were included. All patients were ≥ 18 years and provided written informed consent. Patients who did not switch therapy but received riociguat purely as an add-on to an ERA or prostacyclin analog were not eligible.

### Study Procedures

Data were retrospectively collected from patient medical records for the 12-month period prior to switching and the 5-month period post-switching. For patients who discontinued riociguat, data were still collected for the 5-month post-switch period.

Decisions about clinical management of each patient, including riociguat treatment duration, were determined solely by the treating physician, without influence from the study protocol.

### Outcome Measures

#### Primary Outcome Measures

Primary outcome measures included information on riociguat dose adjustment during switching, vital signs during the dose-adjustment period (systolic and diastolic blood pressure and heart rate), switch medication, reason for switching, and duration of treatment-free periods between previous medication and riociguat.

A treatment-free period was defined as the number of days between the last intake of the switched therapy and the first treatment with riociguat (excluding the last day of pre-switch drug intake and the first day of riociguat treatment). A treatment-free period of 0 indicates that riociguat was started 1 day after the last intake of the switched drug. A negative value for the treatment-free period indicates the switched drug was discontinued after the start of riociguat.

#### Other Variables

Patient characteristics (including baseline demographics, medical history, and clinical characteristics) and clinical parameters (6-min walking distance [6MWD], Borg Dyspnea Index, World Health Organization function class [WHO FC], and biomarkers) were also collected.

#### Safety Assessments

Incidences of adverse events (AEs) and serious adverse events (SAEs), including those of special interest (serious hemoptysis and symptomatic hypotension), were assessed.

### Statistical Analyses

All variables were analyzed descriptively and all patients who received at least one dose of riociguat were included in the analysis. No imputation of missing information was applied except for partial dates (full rules listed in Supplementary Information).

## Results

### Patient Population

**Fig. 1:**
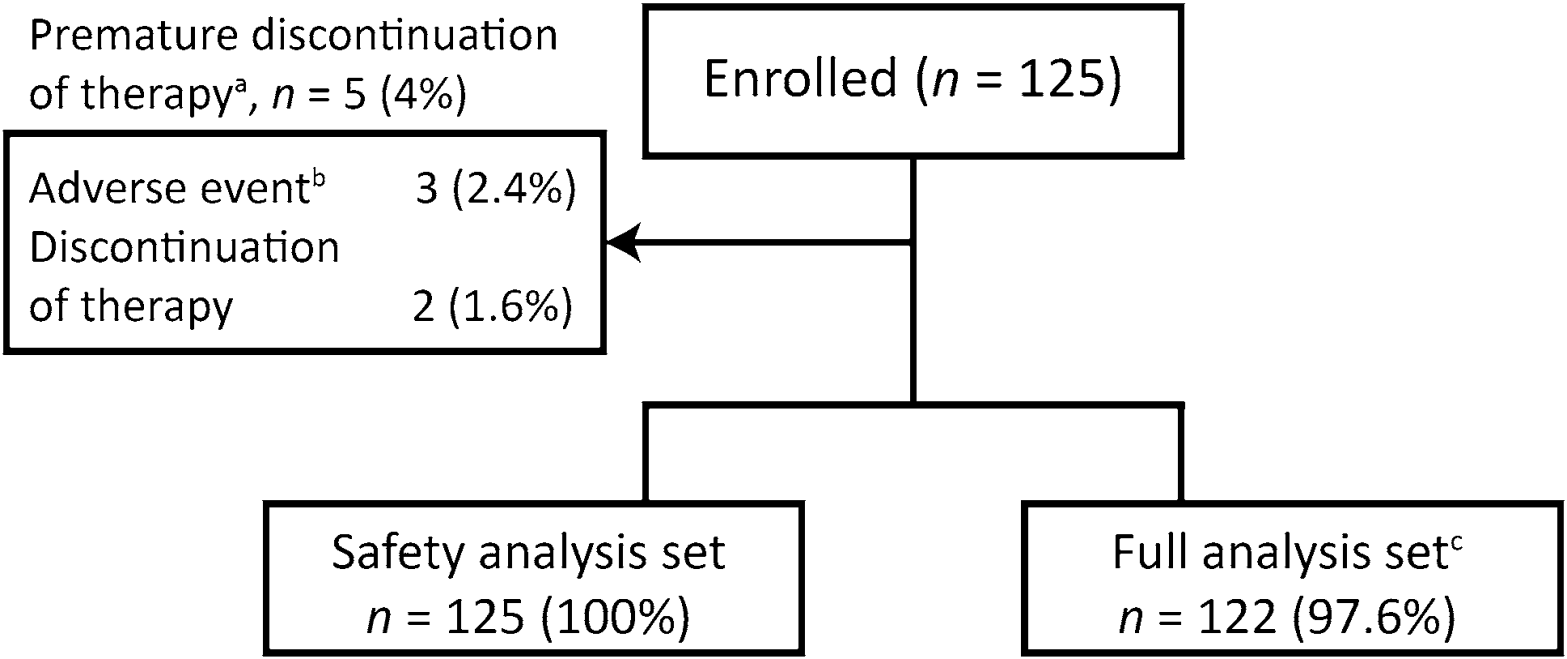
Patient disposition. ^a^Primary reason for premature discontinuation of riociguat therapy. ^b^Adverse events reported: Patient 1–dizziness, dyspepsia, and oxygen consumption increased; patient 2–exacerbation of pulmonary hypertension; patient 3–dyspnea and back pain. ^c^Three patients were not included in the full analysis set due to violation of the inclusion or exclusion criteria

The total documentation period for the study was October 1, 2012 to May 31, 2016. Of the 127 patients screened, 125 were enrolled (Fig. 1). All enrolled patients completed the 5-month post-switch period and were included in the safety analysis set (SAF); 122 (98%) were included in the full analysis set (FAS) due to the exclusion of three patients for violations of the enrollment criteria. FAS data are presented, with the exception of demographic and safety-related data, where SAF data are presented.

At baseline, most patients were in WHO FC III (66%) and had inoperable or persistent/recurrent CTEPH (85%). The mean (standard deviation [SD]) 6MWD and *N*-terminal prohormone of brain natriuretic peptide at baseline were 354 (110) m and 806 (1041) pg/mL, respectively (Table 1).

**Table 1:** Patient and disease characteristics at baseline

Characteristic	Safety analysis set (*n* = 125)
Age, years	64 (16)
Female, *n* (%)	76 (61)
BMI^a^, kg/m^2^	27 (7)
Time from initial diagnosis to start of riociguat, months	55 (54)
PAH, *n* (%)	40 (32)
CTEPH, *n* (%)	85 (68)
Inoperable	33 (39)
Persistent	41 (48)
Recurrent	11 (13)
BPA performed prior to start of riociguat treatment	12 (14)
6MWD^b^, m	354 (110)
WHO FC I/II/III/IV^c^, %	2/27/66/5
NT-proBNP^d^, pg/mL	806 (1041)

Data are mean (SD) unless otherwise stated; ^a^*n* = 111, ^b^*n* = 101, ^c^*n* = 112, ^d^*n* = 47

*6MWD* 6-min walking distance, *BMI* body mass index, *BPA* balloon pulmonary angioplasty, *CTEPH* chronic thromboembolic pulmonary hypertension, *NT-proBNP N*-terminal prohormone of brain natriuretic peptide, *PAH* pulmonary arterial hypertension, *SD* standard deviation, *WHO FC* World Health Organization functional class

Five patients (4%) prematurely discontinued riociguat therapy (Fig. 1).

### Prior Therapy

At the time of switching to riociguat, most patients were receiving PDE5i monotherapy (*n* = 46; 38%) or ERA + PDE5i (*n* = 43; 35%). Most patients switched to riociguat from a PDE5i (*n* = 98, 80%), with 67 (55%) switching from sildenafil and 31 (25%) from tadalafil. The mean (SD) treatment durations prior to switching were 43 (32) and 23 (16) months for sildenafil and tadalafil, respectively.

Among patients with PAH, most were receiving ERA + PDE5i (*n* = 24; 60%) or ERA + PDE5i + prostacyclin (*n* = 9; 23%) at the time of switching, and 34 patients (85%) switched PDE5i for riociguat. Among patients with CTEPH, the most common prior treatment was a PDE5i in 43 patients (52%) and ERA + PDE5i in 19 patients (23%), and 64 patients (78%) switched PDE5i for riociguat (Table 2).

**Table 2:** Duration of treatment-free period during switching (full analysis set)

Switched drug(s)	Treatment-free period^a^, days					
	PAH (*n* = 40)		CTEPH (*n* = 82)		Total population (*n* = 122)	
	*n* (%)	Median (range)	*n* (%)	Median (range)	*n* (%)	Median (range)
PDE5i						
Sildenafil	22 (55)	0 (− 12 to 13)	45 (55)	0 (− 24 to 2)	67 (55)	0 (− 24 to 13)
Tadalafil	12 (30)	2 (0–5)	19 (23)	2 (− 1 to 5)	31 (25)	2 (− 1 to 5)
ERA						
Ambrisentan	0	−	2 (2)	1 (− 1 to 3)	2 (2)	1 (− 1 to 3)
Bosentan	3 (8)	1 (− 1 to 51)	6 (7)	− 1 (− 5 to 16)	9 (7)	− 1 (− 5 to 51)
Prostacyclin analog						
Beraprost	0	−	2 (2)	1 (0–2)	2 (2)	1 (0–2)
Iloprost	1 (3)	0	3 (4)	0 (− 1 to 23)	4 (3)	0 (− 1 to 23)
Double combination therapy						
Bosentan + sildenafil	0	−	2 (2)	6 (− 1 to 13)	2 (2)	6 (− 1 to 13)
Iloprost + sildenafil	1 (3)	3	2 (2)	− 1 (− 1 to 0)	3 (2)	0 (− 1 to 3)
Epoprostenol + sildenafil	1 (3)	0	0	−	1 (1)	0
Triple combination therapy						
Bosentan + iloprost + sildenafil	0	−	1 (1)	− 1	1 (1)	− 1

*CTEPH* chronic thromboembolic pulmonary hypertension, *ERA* endothelin receptor antagonist, *PAH* pulmonary arterial hypertension, *PDE5i* phosphodiesterase type 5 inhibitor

^a^Treatment-free period was the number of days between the day of last intake of switched PH drug(s) and the day of first treatment with riociguat (excluding the last day with pre-switch PH drug intake and the first day with riociguat). If the switched drug was discontinued after the start of riociguat, the treatment-free period is negative. A treatment-free period of 0 indicates riociguat was started 1 day after the last intake of the switched drug

### Post-Switch Therapy

The most common post-switch treatment was riociguat monotherapy (*n* = 64; 52%), or ERA + riociguat (*n* = 45; 37%).

Among patients with PAH, the most common post-switch therapies were ERA + riociguat (*n* = 27; 68%), ERA + riociguat + prostacyclin (*n* = 7; 18%), and riociguat monotherapy (*n* = 6; 15%). Among patients with CTEPH, the most common post-switch therapies were riociguat monotherapy (*n* = 58; 71%) and ERA + riociguat (*n* = 18; 22%).

In line with the contraindication for concomitant use of the two drugs, most patients did not receive riociguat + PDE5i. However, one patient received concomitant riociguat + PDE5i for 12 days. This patient experienced SAEs of worsening right heart failure (two episodes, both of which were resolved and not considered study-drug related); symptomatic hypotension was also reported although not during the time in which concomitant riociguat + PDE5i was administered. The stop date for PDE5i therapy was incomplete for another patient.

### Reason for Switch

The most common reason for switching to riociguat was lack of efficacy of the prior therapy (*n* = 102; 84%). Other reasons included patient request and lack of tolerability (Table 3).

**Table 3:** Reasons for switching to riociguat (full analysis set)

Reasons for switching to riociguat, *n* (%)	PAH (*n* = 40)	CTEPH (*n* = 82)	Total population (*n* = 122)
Lack of efficacy of prior PH-targeted therapy	32 (80)	70 (85)	102 (84)
Patient request	5 (13)	2 (2)	7 (6)
Lack of tolerability	2 (5)	3 (4)	5 (4)
Cost/reimbursement issues	0 (0)	3 (4)	3 (2)
Physician’s decision	1 (3)	2 (2)	3 (2)
Availability of targeted medication	0 (0)	2 (2)	2 (2)

*CTEPH* chronic thromboembolic pulmonary hypertension, *PAH* pulmonary arterial hypertension, *PH* pulmonary hypertension

### Duration of Treatment-Free Period

The median (range) treatment-free period prior to commencing riociguat was 0 (− 24 to 51) days. The median treatment-free period was longer in patients who switched from tadalafil, recorded as 2 (− 1 to 5) days, than in those who switched from sildenafil, recorded as 0 (− 24 to 13) days, where 0 days indicated riociguat was started the day following the last intake of switched drug (Table 2). Due to the study imputation rules for partial or missing dates (see Supplementary Information), the patient with the incomplete stop date (day missing) led to a reported overlap of 24 days, affecting the calculation of the mean treatment-free period for switching from sildenafil to riociguat.

### Dose-Adjustment Procedure

Riociguat was most frequently initiated at 3.0 mg/day (*n* = 79; 65%), followed by 1.5 mg/day (*n* = 20; 16%) and 7.5 mg/day (*n* = 16; 13%); two patients (2%) were started on 1 mg/day. The mean (SD) initial dose was 3.3 (1.8) mg/day.

The mean (SD) number of dose-adjustment steps was 2.4 (1.5), with the majority of patients receiving three or four dose adjustments (*n* = 66; 54% and *n* = 12; 10%, respectively). For 27 patients (22%), only the initial dose was documented. Vital signs were assessed at each dose-adjustment step; mean systolic blood pressure remained above 100 mmHg during each increase in riociguat (Supplementary Table 1).

The mean (SD) maintenance dose of riociguat was 6.7 (1.7) mg/day with 77% of patients achieving a maintenance dose of 7.5 mg/day within 8 weeks.

### Safety

#### Treatment-Free Period

Two patients (2%), both with CTEPH, experienced an AE during the treatment-free period (liver disorder and hyperlipidemia).

#### Dose-Adjustment Period

Most AEs occurred during the 8-week dose-adjustment period (up to Day 56), with 51 patients (41%) experiencing AEs during this time (Table 4, Supplementary Table 2, and Supplementary Fig. 1). The most common AEs were dizziness (*n* = 11; 9%), dyspepsia (*n* = 10; 8%), and headache (*n* = 6; 5%). Hypotension was experienced by five patients (4%).

**Table 4:** Patients experiencing AEs during the dose-adjustment period (safety analysis set)

*n* (%)	Dose-adjustment period^a^ (*n* = 125)
AEs	51 (41)
Drug-related AEs	32 (26)
SAEs	6 (5)
Drug-related SAEs	2 (2)
Discontinuations	1 (1)
AEs resulting in dose reduction or interruption	11 (9)

*AE* adverse event, *SAE* serious adverse event

^a^Data are shown for AEs that start within the dose-adjustment period only, defined as any event arising or worsening on the day of or after start of riociguat where the start date ≤ the date of maintenance dose of riociguat or missing

SAEs occurred in six patients (5%); in two patients (2%) the events were considered study-drug related: one patient experienced palpitations, a viral infection, and cardiac catheterization, and one patient experienced right ventricular failure. One patient (1%) discontinued riociguat during the dose-adjustment period and 11 patients (9%) experienced AEs that resulted in a dose reduction or interruption.

### Efficacy

6MWD, *N*-terminal pro-brain natriuretic peptide, and WHO FC at baseline and last follow-up visit are shown in Supplementary Figs. 2, 3, and 4, respectively.

## Discussion

CAPTURE was a retrospective chart review designed to understand how patients with PAH or CTEPH switched to riociguat from other PH-targeted therapies in real-world clinical practice.

The main reason for switching to riociguat was a lack of efficacy of previous PH-targeted therapies. This is in contrast to previously published studies where patients mainly switched due to comfort, safety, and tolerability [22, 27–36]. However, these studies focused on switching from intravenous or subcutaneous prostacyclins to a second prostacyclin (mainly treprostinil) or an ERA, whereas in CAPTURE, 80% of patients switched from an oral PDE5i. Therefore, as well as the difference in mechanism of action between PH therapies leading to varying side effect profiles, administration procedure may also play a role in the reason for switch. It has also been reported that patients switch from sildenafil due to AEs [21], and in CAPTURE, 4% of the population switched due to lack of tolerability of prior therapy.

In line with ESC/ERS guidelines [1], most patients with PAH were receiving combination therapy before switching; the majority of these switched from a PDE5i to riociguat. However, patients with inoperable or persistent/recurrent CTEPH were receiving off-label PH-targeted therapies, as riociguat is the only medical therapy approved in CTEPH [1, 23, 24]. Following switching, many patients with PAH received double or triple combination therapy in conjunction with riociguat, while most patients with CTEPH switched to riociguat monotherapy.

Of note, due to concomitant riociguat and PDE5i being contraindicated [23, 24], most patients in CAPTURE did not receive riociguat + PDE5i in combination. One patient had overlapping treatment with a PDE5i and riociguat; however, although the patient experienced right heart failure and hypotension after PDE5i had stopped, these events resolved and riociguat treatment was completed.

The US prescribing information recommends 24- and 48-h treatment-free periods for patients switching from sildenafil and tadalafil, respectively [23], based on the drop in systemic blood pressure caused by concomitant administration of PDE5i and nitrates [37, 38], which have a similar mechanism of action to riociguat. The treatment-free periods observed for sildenafil and tadalafil in CAPTURE suggest that clinicians may be in line with the recommendations for sildenafil, but use a longer than recommended treatment-free period for tadalafil.

Riociguat is administered using an 8-week individual dose-adjustment scheme, starting at 1.0 mg three times a day (tid) and is increased every 2 weeks in the absence of hypotension, to a 2.5 mg tid–maximum [23, 24, 39]. CAPTURE showed that physicians tended to adhere to this protocol, with most patients initiating treatment of riociguat at 3 mg/day. However, 24 patients were started at lower than recommended doses of riociguat. This may be due to over-cautiousness on the part of the physician regarding the contraindication and risk of hypotension with PDE5i + riociguat, as 18 of these patients switched from PDE5i therapy. Additionally, 16 patients initiated riociguat at the maximal dose of 7.5 mg/day, which may be due to physician concern for clinical worsening with lower doses.

The percentage of patients receiving a 7.5 mg/day (2.5 mg tid) maintenance dose was similar in CAPTURE to the percentages in the PATENT-1 and CHEST-1 Phase III clinical trials. In CAPTURE, 77% of patients reached the maximum dose by Week 8, while in PATENT-1 and CHEST-1, the maximum dose was reached by 75% (at Week 12) [40] and 77% (at Week 16) [41], respectively.

The data from CAPTURE suggest that switching to riociguat from other PH-targeted therapies in clinical practice may be carried out safely and is well tolerated. AEs were rarely reported during the treatment-free period, and during the dose-adjustment period, only one patient discontinued riociguat, and 11 patients experienced AEs that resulted in dose reduction or interruption. Although it is important to interpret these observations with caution based on the inherent selection bias of a retrospective chart review (discussed below), they are in line with the results of published case studies and retrospective analyses of both PAH and CTEPH patients switching from other PH-targeted therapies (mainly PDE5i) to riociguat in real-world clinical practice [18, 20, 42–45]. The results from CAPTURE also support preliminary data from the RESPITE clinical trial, which indicated that switching from sildenafil or tadalafil to riociguat in patients with PAH not reaching treatment goals was safe and well tolerated [25].

A key limitation of CAPTURE is that, as a retrospective chart review, it had an inherent selection bias. Patients who died after switching but before giving informed consent for the study were not included. Although this bias applies to all chart reviews, it is important to exercise caution when interpreting the data from CAPTURE, as exclusion of patients who died means that the most unwell patients were not included in the safety analyses. Another study limitation, also owing to its retrospective nature, is the limited efficacy data, with only a small proportion of patients having post-baseline measurements for 6MWD, Borg dyspnea index, WHO FC, and biomarkers. These low patient numbers may be reflective of efficacy parameters primarily being assessed in patients who are not responding well to treatment, resulting in biased data. Moreover, clinical parameters were analyzed by original visit number and an artificial visit window scheme, meaning that the data available at each visit were highly variable. These low and variable patient numbers mean that it is almost impossible to interpret the data.

In conclusion, most patients in CAPTURE were initiated and uptitrated on riociguat in line with recommendations in the label, with a similar percentage of patients achieving the maximum maintenance dose in real-world clinical practice as in the PATENT and CHEST clinical trials. No new safety signals were observed. These data suggest that switching may be feasible in the context of current recommendations.

## Electronic supplementary material

Below is the link to the electronic supplementary material.

Supplementary material 1 (DOCX 140 KB)
