# Supplementary material for: Real-World Switching to Riociguat: Management and Practicalities in Patients with PAH and CTEPH
Source: Lung. 2018 Feb 22;196(3):305–12. doi: 10.1007/s00408-018-0100-3 (PMC5942346; doi:10.1007/s00408-018-0100-3)
Supplement: Supplementary file 1 — Supplementary material 1 (DOCX 140 KB) [file 408_2018_100_MOESM1_ESM.docx]

**Supplementary information**

**Fig. 1** Timeline of drug-related AEs experienced by patients throughout the CAPTURE study (SAF)

dose-adjustment period

**Day**

**Number of AEs experienced**

Timeline shows all events with a causal or undeterminable relationship to riociguat. Events presented are those for which a precise start date was recorded

*AE* adverse event

**Fig. 2** 6MWD at baseline and last follow-up visit (FAS)

**6MWD (m)**

**50**

**150**

**100**

**200**

**300**

**250**

**350**

**400**

**0**

**357**

**367**

**Last follow-up visit (n=67)**

**Baseline (n=67)**

Graph shows mean ± standard error of the mean

Data shown are matched pairs, only including patients with data at baseline and last follow-up visit

*6MWD* 6-minute walking distance

**Fig. 3** NT-proBNP at baseline and last follow-up visit (FAS)

**NT-proBNP level (pg/mL)**

**300**

**500**

**400**

**600**

**800**

**700**

**900**

**1000**

**0**

**Baseline (n=29)**

**Last follow-up visit (n=29)**

**756**

**200**

**100**

**713**

Graph shows mean ± standard error of the mean

Data shown are matched pairs, only including patients with data at baseline and last follow-up visit

*NT-proBNP* N-terminal pro-brain natriuretic peptide

**Fig. 4** WHO FC at baseline and last follow-up visit (FAS)

**Proportion of patients (%)**

**30**

**50**

**40**

**60**

**80**

**70**

**90**

**100**

**0**

**Baseline (n=82)**

**Last follow-up visit (n=82)**

**20**

**10**

**24**

**54**

**5**

**5**

**40**

WHO FC IV

WHO FC III

WHO FC II

WHO FC I

**1**

**70**

**1**

Data shown are matched pairs, only including patients with data at baseline and last follow-up visit

Annotations shown are the percentages of patients in each functional class

*WHO FC* World Health Organization functional class

**Table 1** Systolic blood pressure, diastolic blood pressure, and heart rate at individual titration steps of the dose-adjustment period (full analysis set)

| **Titration step** | | **Systolic blood pressure**  **(mmHg)** | | | **Diastolic blood pressure**  **(mmHg)** | | | **Heart rate**  **(bpm)** | |
| --- | --- | --- | --- | --- | --- | --- | --- | --- | --- |
|  | *N* | | Mean (SD) | *n* | | Mean (SD) | *n* | | Mean (SD) |
| Initial dose | 120 | | 122.1 (19.8) | 120 | | 72.2 (11.6) | 120 | | 77.5 (12.8) |
| 1 | 76 | | 119.7 (19.4) | 75 | | 70.4 (11.1) | 63 | | 79.1 (14.5) |
| 2 | 73 | | 119.4 (18.5) | 73 | | 69.2 (12.3) | 64 | | 78.7 (13.9) |
| 3 | 69 | | 115.5 (16.8) | 69 | | 68.8 (11.6) | 56 | | 81.7 (14.0) |
| 4 | 16 | | 117.8 (17.0) | 16 | | 65.2 (9.4) | 16 | | 74.6 (7.8) |
| 5 | 4 | | 109.5 (9.9) | 4 | | 69.0 (6.2) | 4 | | 74.5 (16.2) |
| 6 | 1 | | 115.0 | 1 | | 74.0 | 1 | | 60.0 |
| 7 | 2 | | 116.0 (1.4) | 2 | | 65.0 (11.3) | 2 | | 76.5 (19.1) |
| 8 | 1 | | 133.0 | 1 | | 76.0 | 1 | | 69.0 |

*bpm* beats per minute, *SD* standard deviation

**Table 2** AEs and SAEs occurring during the 8-week dose-adjustment period (SAF)

|  | **No. of patients, n (%)** |
| --- | --- |
| **AEs^a^**  Dizziness | 11 (9) |
| Dyspepsia | 10 (8) |
| Headache | 6 (5) |
| Dyspnea | 5 (4) |
| Hypotension | 5 (4) |
| Edema | 4 (3) |
| Nasal congestion | 4 (3) |
| Vertigo | 4 (3) |
| Cough | 3 (2) |
| Edema (peripheral) | 3 (2) |
| Nasopharyngitis  **SAEs**  Atrial tachycardia  Cardiac catheterization | 3 (2)  1 (1)  1 (1) |
| Cardiac failure | 1 (1) |
| Palpitations | 1 (1) |
| Polyarthritis  Right ventricle failure | 1 (1)  1 (1) |
| Uterine hemorrhage | 1 (1) |
| Viral infection | 1 (1) |

Patients could report more than one AE or SAE

^a^Occurring in >2% of patients

*AE* adverse event, *SAE* serious adverse event.

**Handling of missing data**

No imputation of missing information was applied except for partial dates. If only partial dates were available the following general data handling rules were used for adverse events, prior and concomitant medications and medical history.

If only the day was missing and the month was available the following imputation rules were applied:

1. In case only the start day was missing, the date was imputed as the first day of the month. For adverse events the date was imputed as the maximum of the start date of riociguat and the day of the incomplete date replaced by the first day of the month.
2. In case only the stop day was missing, the date was imputed as a minimum of date of last follow-up visit and the day of the incomplete date replaced by the last day of the month.

If both the day and the month were missing, the date was considered missing with the exception of prior and concomitant medication, date of initial diagnosis, and medical history where the following imputation rules will be applied:

1. In case that the start day and month were missing, i.e. only the year was available, the day and month were imputed by January 1st.
2. For concomitant medication: In cases where the start date was missing completely and parts of the stop date were available or medication was ticked as ongoing, the start date was replaced with the minimum of January 1st of the year of first riociguat intake and the concomitant medication stop date, since information was available that the patient took previous or concomitant medication and it could be assumed they were on the medication already at the start of the study.
3. For medical history: In cases where the start date was missing completely and parts of the stop date were available or disease was ticked as ongoing, the start date was replaced with the minimum of January 1st of the year of first riociguat intake and the medical history stop date, since information was available that the documented medical history was prior or concomitant and it could be assumed the disease was already present at the start of the study.
4. In case the stop day and month were missing, i.e. only the year was available, the date was imputed as a minimum of date of last follow-up visit and the day and month of incomplete date replaced by December 31st.
5. In case the medication/finding stop date was missing completely and the medication/finding was ticked as ongoing the date was imputed as date of last follow-up visit.
